# Predicting Anticancer Drug Response With Deep Learning Constrained by Signaling Pathways

**DOI:** 10.3389/fbinf.2021.639349

**Published:** 2021-04-29

**Authors:** Heming Zhang, Yixin Chen, Fuhai Li

**Affiliations:** ^1^ Department of Computer Science, Washington University in St. Louis, St. Louis, MO, United States; ^2^ Institute for Informatics, Washington University School of Medicine, St. Louis, MO, United States; ^3^ Department of Pediatrics, Washington University School of Medicine, Washington University in St. Louis, St. Louis, MO, United States

**Keywords:** precision medicine, mechanism of response, cancer, artificial intelligence, deep learning, drug response prediction, signaling pathways

## Abstract

Thanks to the availability of multiomics data of individual cancer patients, precision medicine or personalized medicine is becoming a promising treatment for individual cancer patients. However, the association patterns, that is, the mechanism of response (MoR) between large-scale multiomics features and drug response are complex and heterogeneous and remain unclear. Although there are existing computational models for predicting drug response using the high-dimensional multiomics features, it remains challenging to uncover the complex molecular mechanism of drug responses. To reduce the number of predictors/features and make the model more interpretable, in this study, 46 signaling pathways were used to build a deep learning model constrained by signaling pathways, consDeepSignaling, for anti–drug response prediction. Multiomics data, like gene expression and copy number variation, of individual genes can be integrated naturally in this model. The signaling pathway–constrained deep learning model was evaluated using the multiomics data of ∼1000 cancer cell lines in the Broad Institute Cancer Cell Line Encyclopedia (CCLE) database and the corresponding drug–cancer cell line response data set in the Genomics of Drug Sensitivity in Cancer (GDSC) database. The evaluation results showed that the proposed model outperformed the existing deep neural network models. Also, the model interpretation analysis indicated the distinctive patterns of importance of signaling pathways in anticancer drug response prediction.

## Introduction

Precision medicine or personalized medicine is becoming feasible and widely adopted in cancer treatment due to the availability of multiomics data that comprehensively characterize individual cancer samples. For example, comprehensive multiomics data, like gene expression, copy number variation (CNV), genetic mutation, methylation, and proteomics, as well as clinical outcome information of over 20,000 cancer patients across 33 cancer types and subtypes are available in the cancer genome atlas (TCGA) program (Goldman et al., 2018). On the other hand, the cancer cell lines are important experimental models for evaluating important biomarkers and screening effective drugs in laboratories. The comprehensive multiomics data of >1,000 cancer cell lines were generated and available in the Broad Institute Cancer Cell Line Encyclopedia (CCLE) database (Barretina et al., 2012; Wang et al., 2019). In addition, the drug response of ∼1,000 cancer cell lines against ∼100 drugs and compounds is available in the Genomics of Drug Sensitivity in Cancer (GDSC) database (Garnett et al., 2012; Yang et al., 2013), with the aim of uncovering the potential associations between genetic biomarkers and drug response. Also, about 5,232 drug combination screening of 104 drugs against 60 cancer cell lines are available in the NCI-ALMANAC Drug Combination database (Holbeck et al., 2017). These valuable data sets provide a basis for fully understanding the potential molecular mechanism of cancer heterogeneity and diversity, as well as for understanding the potential mechanism of response (MoR) to anticancer drug treatments.

However, it remains challenging to integrate and interpret the diverse and large number of data points in the high-dimensional multiomics data in a biologically meaningful manner. Though associations between individual biomarkers and drug response have been identified, it is still challenging to decode and uncover the complex signaling networks (interactions of a group of molecules to control specific cellular functions) that regulate the anticancer drug response based on the high-dimensional multiomics data sets. For example, an elastic net model was employed in CCLE and GDSC data analysis to associate the individual biomarkers to the drug response (Barretina et al., 2012; Garnett et al., 2012; Yang et al., 2013; Wang et al., 2019). A support vector machine (SVM)–based model was proposed to predict drug response based on chemical structures and multiomics-based cancer cell line similarity (Wang et al., 2016). Also, drug–cancer cell line similarity–based network models (Sheng et al., 2015; Zhang et al., 2015) were proposed to predict the drug response, and the recommender system was used for drug response prediction (Suphavilai et al., 2018).

Deep learning models have also been proposed for drug response prediction. For example, chemical structure features and omics data have been used as the input of an auto-encoder to reduce the dimension of features and then predict the drug response using deep neural network (DNN) (Li et al., 2019). In addition, a DNN framework was developed to predict anticancer drug response based on gene expression data (Sakellaropoulos et al., 2019), and the results showed that the DNN models outperformed the current machine learning frameworks. Similar models were also proposed to predict drug combination response to anticancer drugs. For example, DeepSynergy (Preuer et al., 2018) and AuDNNsynergy (Zhang et al., 2018) were proposed by integrating chemical structure and genomics features of cancer cell lines and cancer patients in auto-encoders. To understand the mechanism of model prediction in the “black boxes” of deep learning models, the visible neural network (VNN) models (e.g., DCell and DrugCell) (Yu et al., 2018; Kong et al., 2020; Kuenzi et al., 2020) were proposed, using large hierarchical deep learning architecture to model the hierarchical organization of biological processes and to predict drug response with important biomarkers. In DrugCell, large-scale omics data and chemical structure data were used. However, the pathway-level activity was not specifically investigated. Moreover, PASNet (Hao et al., 2018) and Path-DNN (Deng et al., 2020) were proposed to incorporate biological network information. In PASNet, cancer patients’ survival was predicted based on gene expression data. In Path-DNN (Deng et al., 2020), the gene expression data of a set of landmark genes and all general KEGG pathways were used to predict drug response.

In this study, we aimed to improve these models, by developing a deep learning model constrained by signaling pathways, consDeepSignaling, which investigated the activity of 46 signaling pathways (the 45 pathways named with signaling pathways plus the cell cycle pathway) collected from the KEGG signaling pathway database (Ogata et al., 1999; Kanehisa and Goto, 2000; Feng et al., 2020; Zhang et al., 2020) using both the gene expression and copy number variation data of individual genes. Besides that, we leveraged the powerful tool to interpret our models in a global view, which will facilitate the study of signaling pathways in the biomedicine field. In cancer studies, signaling pathways are important concepts that define the signaling cascades among a set of gene/proteins and are therefore biologically meaningful and interpretable to explain the drug response (Sanchez-Vega et al., 2018). For example, the 10 major cancer-related signaling pathways were analyzed using the comprehensive multiomics data of TCGA cancer samples (Sanchez-Vega et al., 2018). The analysis results indicated that about 89% of cancer samples had at least one driver alteration among these signaling pathways. Therefore, we aim to investigate the possibility of using a deep learning model constrained by 46 signaling pathways to predict anticancer drug response. The proposed model was evaluated and compared with existing models using the omics data of cancer cell lines in CCLE and drug response data in the GDSC data set.

## Materials and Methodology

### Genomics of Drug Sensitivity in Cancer Fitted Dose Response Data and Multi-Omics Data

The drug–dose response and omics data of cancer cell lines were obtained from the GDSC database (Table 1). From this data set, the area under the experimental dose–response curve of a given cancer cell line was used to indicate drug effects on the cancer cell line, with a 3-element tuple: < $D_{A},\;C_{B},\;AUC_{AB}$ >. We selected the cancer cell lines that had both gene expression and copy number data. For gene selection, we filtered out those genes which had over 1/3 zero values (missing data) in all cell lines and selected the genes with both gene expression and copy number data. Finally, we obtained 929 gene RNA sequence data (rnaseq_fpkm) and copy number data (cnv_gistic) of 791 cancer cell lines.

**TABLE 1:** Data sets collected from public GDSC database.

Data set	Link
GDSC dose response	http://ftp.sanger.ac.uk/pub/project/cancerrxgene/releases/current_release/GDSC2_fitted_dose_response_25Feb20.xlsx
GDSC RNAseq	https://cog.sanger.ac.uk/cmp/download/rnaseq_20191101.zip
GDSC copy number variation	https://cog.sanger.ac.uk/cmp/download/cnv_20191101.zip

### Drug–Target Information

There are 54 common drugs between DrugBank (Wishart et al., 2018) and GDSC data set. The drug target information was derived from DrugBank database; and 24 out of the 54 drugs were selected for this study, whose target genes are on the 46 signaling pathways (see Table 2).

**TABLE 2:** Twenty-four drugs used in the proposed model.

Afatinib	Crizotinib	Dabrafenib	Daporinad	Dasatinib	Erlotinib
Gefitinib	Lapatinib	Leflunomide	Linsitinib	MK-1775	Navitoclax
Nilotinib	Osimertinib	Palbociclib	Ribociclib	Ruxolitinib	Sorafenib
Staurosporine	Tamoxifen	Trametinib	Ulixertinib	Vinblastine	Vorinostat

### Kyoto Encyclopedia of Genes and Genomes (KEGG) Signaling Pathways

In the KEGG signaling database, 46 signaling pathways were collected (the 45 pathways named with signaling pathways plus the cell cycle pathway). These signaling pathways were MAPK, FoxO, TGF-beta, ErbB, VEGF, Ras, Rap1, p53, Hippo, TNF, mTOR, PI3K-Akt, estrogen, NF-kappa B, notch, JAK-STAT, Wnt, hedgehog, HIF-1, T-cell receptor, adipocytokine, sphingolipid, B-cell receptor, oxytocin, phospholipase D, apelin, Fc epsilon RI, glucagon, relaxin, calcium, toll-like receptor, neurotrophin, AGE-RAGE, cGMP-PKG, NOD-like receptor, insulin, cell cycle, cAMP, AMPK, RIG-I–like receptor, GnRH chemokine, C-type lectin receptor, prolactin, IL-17, and thyroid hormone. There were 929 genes that had both gene expression and copy number variation data in these 46 signaling pathways.

### The ConsDeepSignaling Model

**FIGURE 1:**
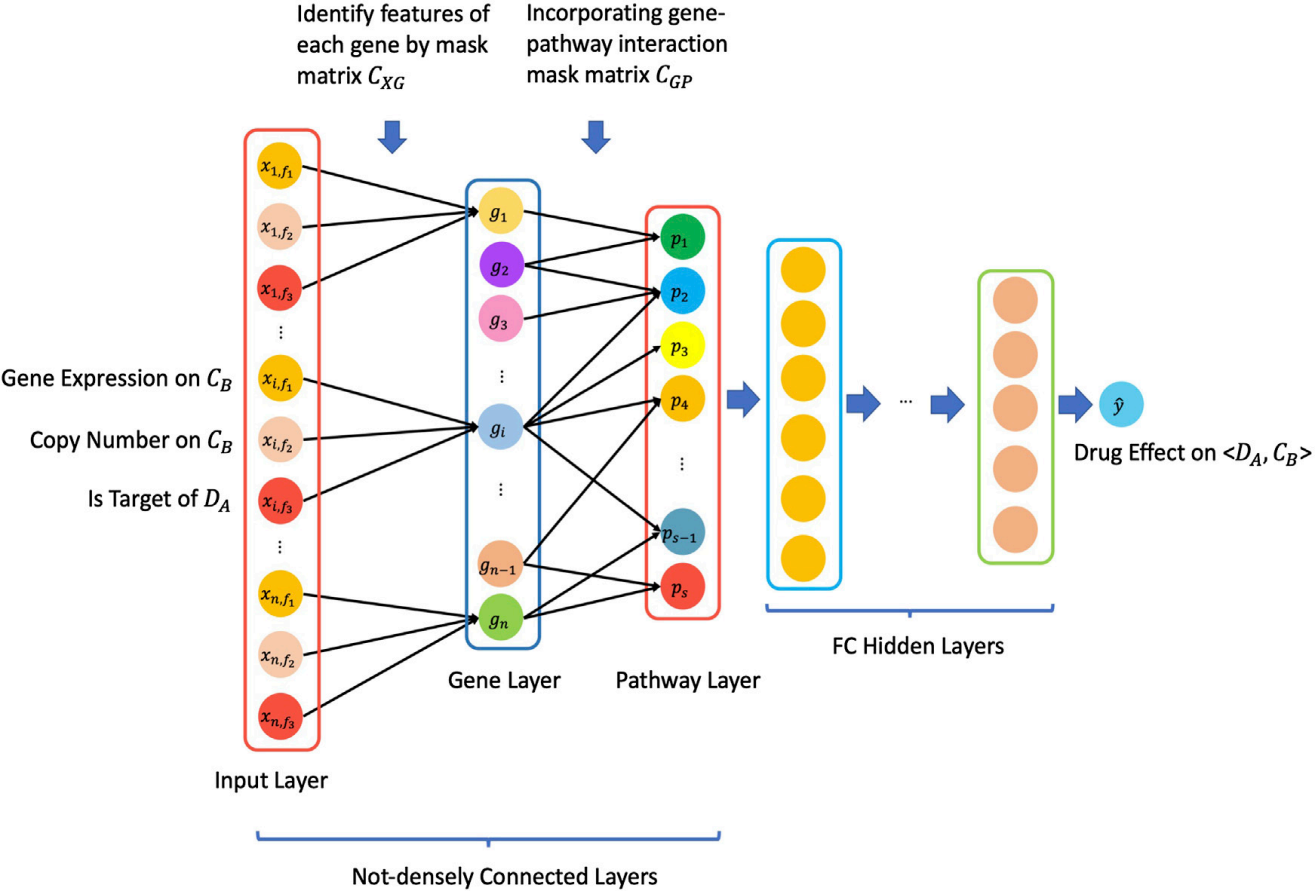
Schematic architecture of the proposed *consDeepSignaling* model.

Figure 1 illustrated the overview architecture of the consDeepSignaling model. We denote the input layer node vector with by $X={\left[x_{1,f_{1}},x_{1,f_{2}},\ldots x_{1,f_{K}},\ldots ,x_{i,f_{k}}\ldots ,x_{n,f_{K}}\right]}^{\text{T}}\in {\mathbb{R}}^{Kn\times 1},\;$ where K equals the number of features for each gene, n equals the number of genes, and $x_{i,f_{k}}$ denotes the k th feature of i th gene. For each gene, we have K features. Therefore, one set of gene features can be denoted as $x_{i}=\left(x_{i,f_{1}},x_{i,f_{2}},\ldots ,x_{i,f_{K}}\right)\in {\mathbb{R}}^{K\times 1}$ . Then, we have $X=\left(x_{1}\left|x_{2}\right|\ldots |x_{i}|\ldots |x_{n}\right)\;\in {\mathbb{R}}^{Kn\times 1}$ . We denote the gene layer node vector by $G=\left(g_{1},g_{2},\ldots ,g_{i}\ldots ,g_{n}\right)\in {\mathbb{R}}^{n\times 1}$ , where n equals the number of genes and $g_{i}$ denotes i th gene. Thus, we denote the weight parameter between X and G with matrix $W_{XG}\in {\mathbb{R}}^{Kn\times n}$ . Since we only make connection between certain gene features and their corresponding genes, we will create a gene features connection matrix with $C_{XG}\in {\mathbb{R}}^{Kn\times n}$ . The elements $c_{i,j},\;\left(i=1,2,\ldots ,Kn,\;j=1,2,\ldots ,n\right),\;$ in this matrix follow the rule that $c_{i,j}=1,\;$ for i=K(j−1)+m, (m=1,2,…,K). Then, the matrix $C_{XG}\in {\mathbb{R}}^{Kn\times n}$ is as follows.

$$C_{XG}=\left[\begin{array}{ccc} \begin{array}{cc} c_{1,1} & 0\\ \vdots & \vdots \\ c_{K,1} & 0 \end{array} & \cdots & \begin{array}{c} 0\\ \vdots \\ 0 \end{array}\\ \begin{array}{cc} \vdots & \vdots \end{array} & \ddots & \vdots \\ \begin{array}{cc} 0 & 0\\ \vdots & \vdots \\ 0 & 0 \end{array} & \cdots & \begin{array}{c} c_{Kn-K+1,n}\\ \vdots \\ c_{Kn,n} \end{array} \end{array}\right]$$

and then we use matrix $C_{XG}$ as a mask matrix to forward information from the input layer to gene layer by

$${\left(C_{XG}\;\cdot W_{XG}\right)}^{\text{T}}X=G$$

Similarly, the pathway layer is denoted by $P=\left(p_{1},p_{2},\ldots ,p_{i},\ldots ,p_{s}\right)\in {\mathbb{R}}^{s\times 1}$ , where s equals the number of signaling pathways. Thus, we denote the weight parameters between G and P using the matrix $W_{GP}\in {\mathbb{R}}^{n\times s}$ . Here, we will use the gene–pathway connection matrix $C_{GP}\in {\mathbb{R}}^{n\times s}$ to demonstrate the connection. Therefore, we use matrix $C_{GP}$ as a mask matrix to forward information from the gene layer to pathway layer by

$${\left(C_{GP}\cdot W_{GP}\right)}^{\text{T}}G=P$$

From the pathway layer to the output layer, all of the layers are fully connected to obtain a scalar with a value which is our prediction $\hat{y}$ .

For this specific study, in the “input layer,” there were 3 input features, that is, gene expression (rnaseq_fpkm), copy number (cnv_gistic), and is_target_of_Drug (0: this gene is not a target of a drug; 1: this gene is a target of a drug), for each of 929 genes on cancer cell lines. Therefore, we have $f_{1}$ for gene expression, $f_{2}$ for copy number, and $f_{3}$ for is_target_of_Drug; and here, K=3 and n=929 . There were 929 genes connected with the 46 signaling pathways (denoted by using the weight matrix $W_{GP}$ ). Hence, we get s=46 . (See the Supplementary Table S1, TS1_drug_gene_pathway.csv). The output of the “46 signaling pathways” was used as the input of the deep belief network (DBN) (densely connected). The output layer was the predicted AUC value of drug effect $D_{A}$ on cancer cell line $C_{B}$ . The mean square error (MSE) was used as the loss function. For the DBN, there were 3 hidden layers, which were 3^rd^, 4^th^, and 5^th^ hidden layers in the whole model: 3^rd^ hidden layer had 256 nodes with the ReLU activation function; the 4^th^ hidden layer had 128 nodes with the ReLU activation function; and the 5^th^ hidden layer had 32 nodes with the ReLU activation function. The linear activation function was used in the output layer. At last, to investigate the importance of individual signaling pathways to the drug response prediction, the SmoothGrad model in the “iNNvestigate” package (Alber et al., 2018) was employed to interpret the deep learning model at the pathway layer. SmoothGrad (Smilkov et al., 2017) was developed to extract the gradients from the trained model to indicate the feature importance. The code and data are available at https://github.com/SynergisticDrugCombinationPrediction/ConsDeepSignaling.

## Experiment Results

**FIGURE 2:**
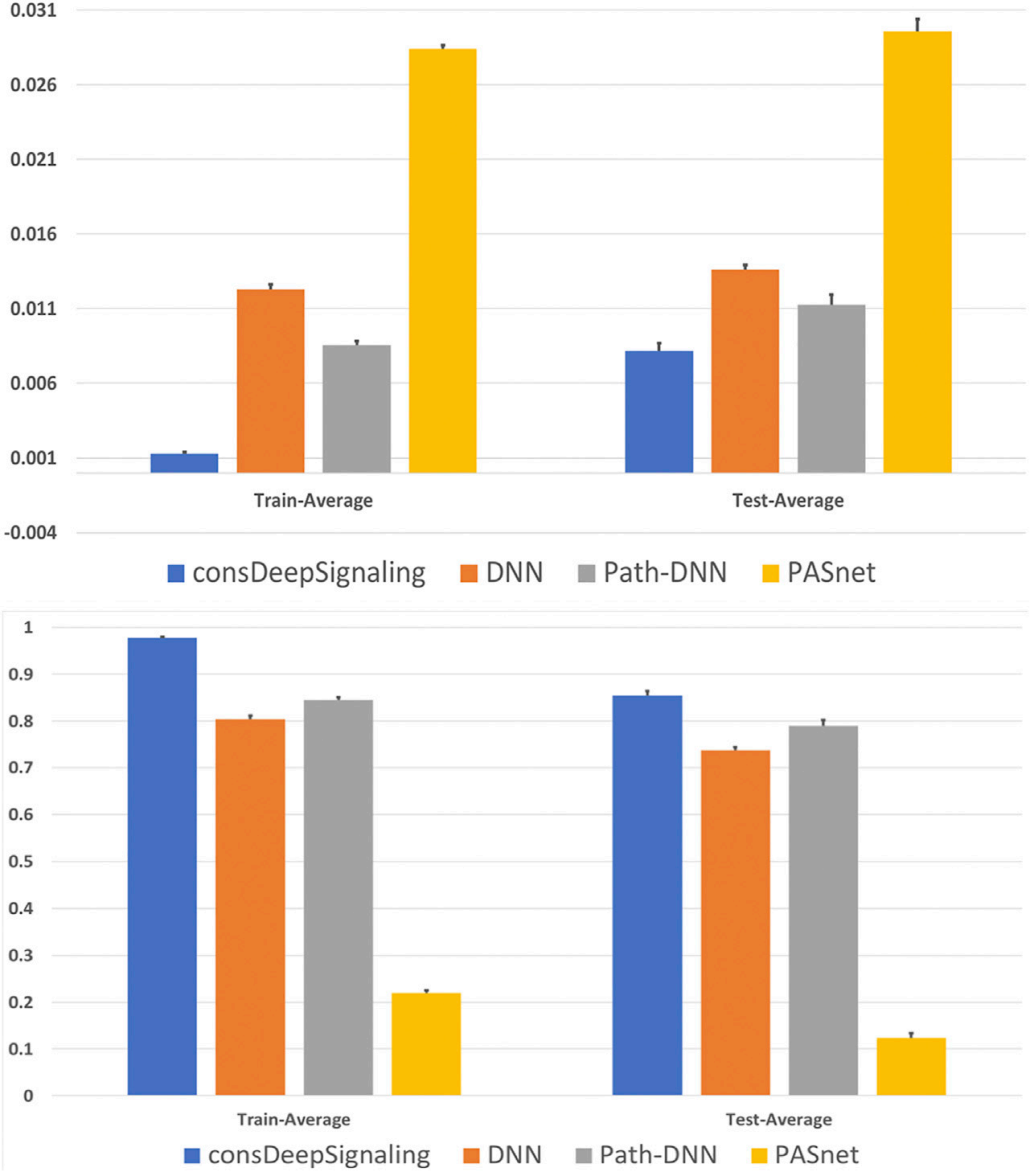
Average MSE loss **(upper panel)** and Pearson correlation coefficients **(bottom panel)** on the 5 training and testing data sets of four models.

**FIGURE 3:**
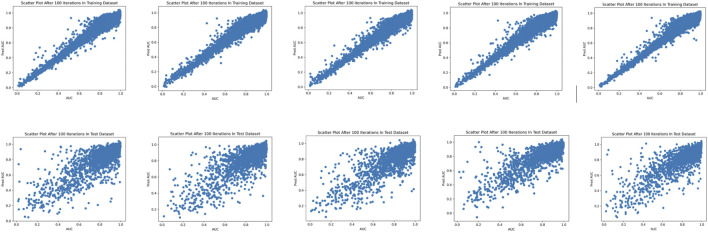
Scatterplots of the predicted and experimental drug response scores on the five-fold training **(upper panel)** and test **(bottom panel)** data sets.

To evaluate the performance of the proposed consDeepSignaling model, the drug responses of 24 drugs on the 791 cancer cell lines collected data from the GDSC database were used. For all of the following models, we leveraged five-fold cross-validation. In the 1st to 4th splits of training and test data sets, it contained 13,409 and 3,352 points, respectively, and in the 5th split of training and test data set, it contained 13,408 and 3,353 points. For the proposed model, to avoid oscillation, we used varying learning rate schedule to adjust the learning rate in different stages of epoch: for 1–30, 31–40, 41–50, 51–70, and 71–100 epochs, the learning rates $1\times 10^{-3}$ , $1\times 10^{-4}$ , $5\times 10^{-5}$ , $1\times 10^{-5}$ , and $1\times 10^{-6}$ were used, respectively. Figure 2 shows the MSE Loss and the Pearson correlation coefficients on the five-fold cross-validation training and validation data sets using the consDeepSignaling model. On average, the proposed consDeep Signaling achieved about 0.98 and 0.85 Pearson correlation coefficients on the training and testing data sets on the five-fold cross-validation, respectively. Figure 3 showed the scatterplots of the training (upper panel) and test (bottom panel) data sets of the five-fold cross-validation, respectively.

We further compared the proposed model with the deep neural network (DNN) model. The input layer still contained the same information as the proposed model. For 1st and 2nd hidden layers, we set 256 nodes in each layer, and we used fully connected layers with LeakyReLU as activation functions by setting the parameter α=0.3 and the dropout rate with 0.1. For the 3rd, 4th, and 5th fully connected hidden layers, we set 256, 128, and 32 nodes in the DNN model. To avoid oscillation, we used varying learning rate schedules to adjust the learning rate in different stages of epoch: for 1–15, 16–30, 31–50, 51–70, and 71–100 epochs, the learning rates $1\times 10^{-3}$ , $1\times 10^{-4}$ , $5\times 10^{-5}$ , $1\times 10^{-5}$ , and $1\times 10^{-6}$ were used, respectively. The DNN model achieved about 0.80 and 0.74 for Pearson correlation coefficients on the training and testing data sets of five-fold cross-validation, which are lower than our proposed model. Moreover, we also compared our model with pathway-guided models. In Path-DNN, we used gene expression data and drug targets information in the input layer for 929 genes. Then, we used corresponding gene–pathway connection matrix to forward information from the input layer to the 46-pathway layer. From the pathway layer to the output layer, we used two fully connected layers with 512 and 32 nodes with ReLU as activation function. The linear activation function was used in the output layer to make prediction. In PASNet, we used gene expression data in the input layer for 929 genes, connected to the 46-pathway layer. From the pathway layer to the output layer, there was only one hidden layer in PASNet. In the hidden layer, the ReLU function was implemented as the activation function with the dropout rate of 0.1. For the output layer, the activation function was the linear function. Both the hidden layer and the output layer used the L2 regulaizer with the parameter as 0.01. The 5-fold cross-validation test results for all aforementioned four models (consDeepSignaling, DNN, Path-DNN and PASNet) can be found in Supplementary Tables S2–S5 (TS. 2–5, respectively). Figure 2 shows the averaged value of MSE loss (upper panel) and Pearson correlation coefficients (bottom panel) on the 5 training and testing data sets of the four models. As seen, the proposed model outperformed the other models. We also tested the consDeepSignaling predictions using the 10 sets of randomly permuted labels of the samples to simulate the random prediction. The average Pearson correlation coefficients on the randomly permutated training and testing data sets were very low, that is, 0.24 and 0.001. The p-values and FDRs of observing the Pearson correlation coefficients on the real testing data sets are all 0, calculated using the empirical cumulative distribution function estimated using the Pearson correlation coefficients of the randomly permutated data sets, which indicate that the predictive models are much reliable than random prediction.

**FIGURE 4:**
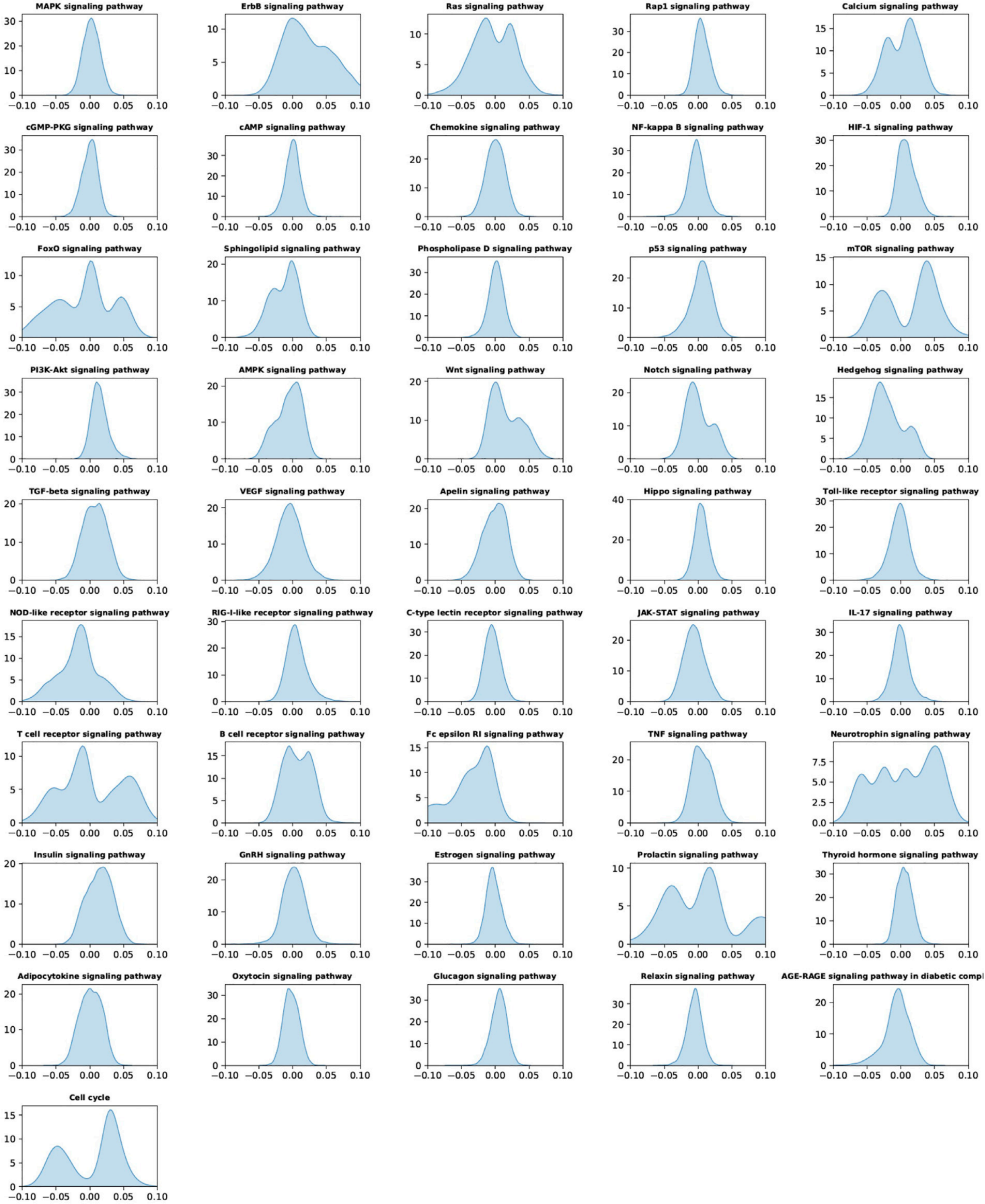
Importance of score distribution patterns of the 46 signaling pathways in all samples by pooling all the testing data sets of the five-fold cross-validation.

To understand the predictive importance of individual signaling pathways and drug response, the “SmoothGrad” model in the “iNNvestigate” package was employed to interpret the deep learning model at the signaling pathway layer. Figure 4 showed the importance score distributions of individual signaling pathways of all the samples, by pooling the importance scores of individual signaling pathways in all samples in the 5 testing data sets. The important distributions were a global analysis of the signaling pathways using all the samples. As seen, compared with other signaling pathways, the ErbB, Ras, Calcium, FoxO, mTOR, Wnt, hedgehog, NOD-like receptor, T-cell receptor, Fc epsilon RI, neurotrophin, insulin, prolactin, and cell cycle signaling pathways have much larger range of importance scores, which indicated the importance of these signaling pathways in anticancer drug response prediction.

## Discussion and Conclusion

Large-scale and high-dimensional multiomics data, like gene expression, copy number variation, methylation, genetic mutation, and microRNA, of individual cancer patients and >1,000 cancer cell lines have been generated, which provide the basis to fully understand the molecular mechanisms of tumor heterogeneity and diversity, as well as the heterogeneous response to anticancer drugs. It also enables precision medicine or personalized medicine selects the right and optimal treatments for individual patients based on their omics profiles and biomarkers. A few databases have been generated and publicly available to access these valuable data resources, like CCLE (Barretina et al., 2012; Wang et al., 2019), and GDSC (Garnett et al., 2012; Yang et al., 2013).

Though the machine learning– and deep learning–based association studies have been reported to identify potential biomarkers correlating with distinct drug response, it remains challenging to integrate the large-scale and high-dimensional data features in a biologically meaningful manner and to further decode and uncover the mysterious molecular mechanisms of drug response for the purpose of precision medicine. In this study, we proposed a novel deep learning model–constrained integrating gene expression and copy number data constrained by signaling pathways, consDeep signaling, to model the pathway activity and their capacity to predict the drug response. The advantages of the consDeepSignaling are 1) it used a small set of genes and modeled the gene activity using multiomics data; 2) it used only 46 signaling pathways to predict the drug response, which has a smaller number of parameters than the existing DNN models; 3) the model was more interpretable; and 4) the model interpretation analysis was conducted to identify the potentially important signaling pathways to inhibit the tumor growth. The evaluation and comparison results indicated that the proposed model outperformed the existing models.

It is an exploratory study to integrate biologically meaningful signaling pathways with the deep learning for anticancer drug response on the 791 cancer cell lines. There are some limitations to be further investigated. For example, though it might be challenging, further functional analysis on the important signaling pathways of individual cancer cell lines might be able to identify the specific cancer cell line–specific dysfunctional targets that are responsible for drug response. Also, in addition to the signaling pathways, more biological processes (BPs), for example, the gene ontologies (GOs) (Ashburner et al., 2000) should be considered. Then, more drugs whose targets are not on the 46 signaling pathways could be included. Moreover, the graph neural network (GNN) model might be able to model the signaling cascades directly to identify the unknown molecular mechanisms (in terms of signaling network modules) that are responsible for the drug response. We will investigate these challenges in future work. In the analysis, it is assumed that drugs with the same targets should have similar effects. The status of target genes will be decided (learned by the model) by the gene expression, copy number, and if it is a drug target (not drug-specific). Then, the status of the target genes will affect (parameters to be learned by the model) the pathway activities to influence the drug response prediction. It is interesting to study the drug-specific effects on the target genes. Moreover, different drugs and cell lines could have drug- and cell-specific signaling pathways that are informative for the drug response prediction. Thus, it is interesting and important to investigate the drug- and cell-specific pathway activity as well as identify the potentially important genes that can affect the activity of individual signaling pathways. As seen in the results, a larger difference in performance between the training and test sets in the proposed model, than other models, indicated the overfitting on the training data sets. We will systematically evaluate some widely used techniques to reduce the overfitting problem, for example, early stopping, adding noise to the input data, and adding a penalty term to control the nonzero parameters.

## Data Availability

Publicly available data sets were analyzed in this study. This data can be found here: The drug response data can be found at the GDSC database: https://www.cancerrxgene.org. The multiomics data of cancer cell lines can be found at the CCLE database: https://portals.broadinstitute.org/ccle. The signaling pathway information can be found at the KEGG signaling pathway database: https://www.genome.jp/kegg/pathway.html. The drug-target information can be found at the drugBank database: https://go.drugbank.com/.
