# Supplementary material for: Predicting Anticancer Drug Response With Deep Learning Constrained by Signaling Pathways
Source: Front Bioinform. 2021 Apr 29;1:639349. doi: 10.3389/fbinf.2021.639349 (PMC9581064; doi:10.3389/fbinf.2021.639349)

FS.1 Importance scores of the 46 signaling pathways in 1^st^ split of testing dataset of the 5-fold cross-validation.


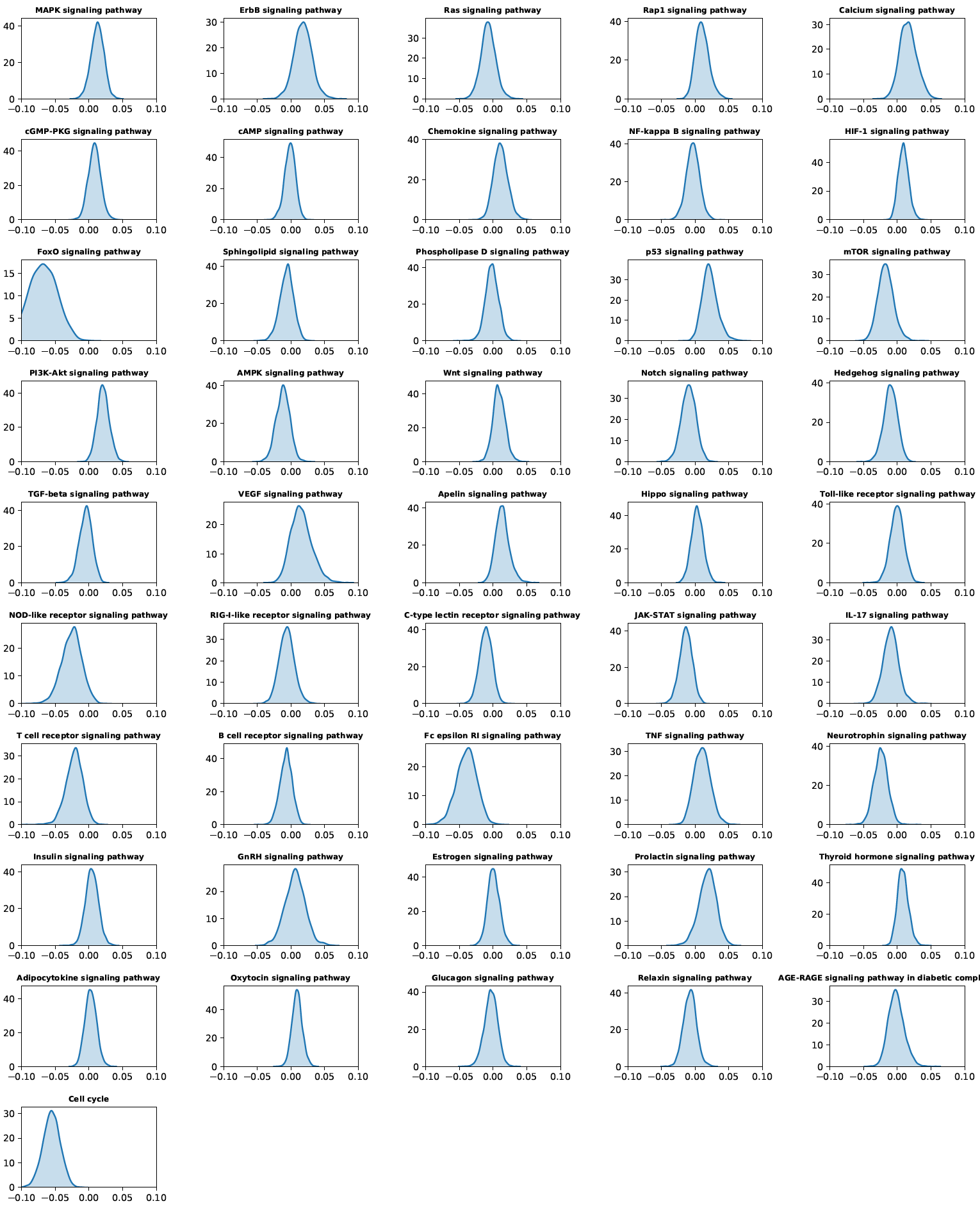


FS.2 Importance scores of the 46 signaling pathways in 2^nd^ split of testing dataset of the 5-fold cross-validation.


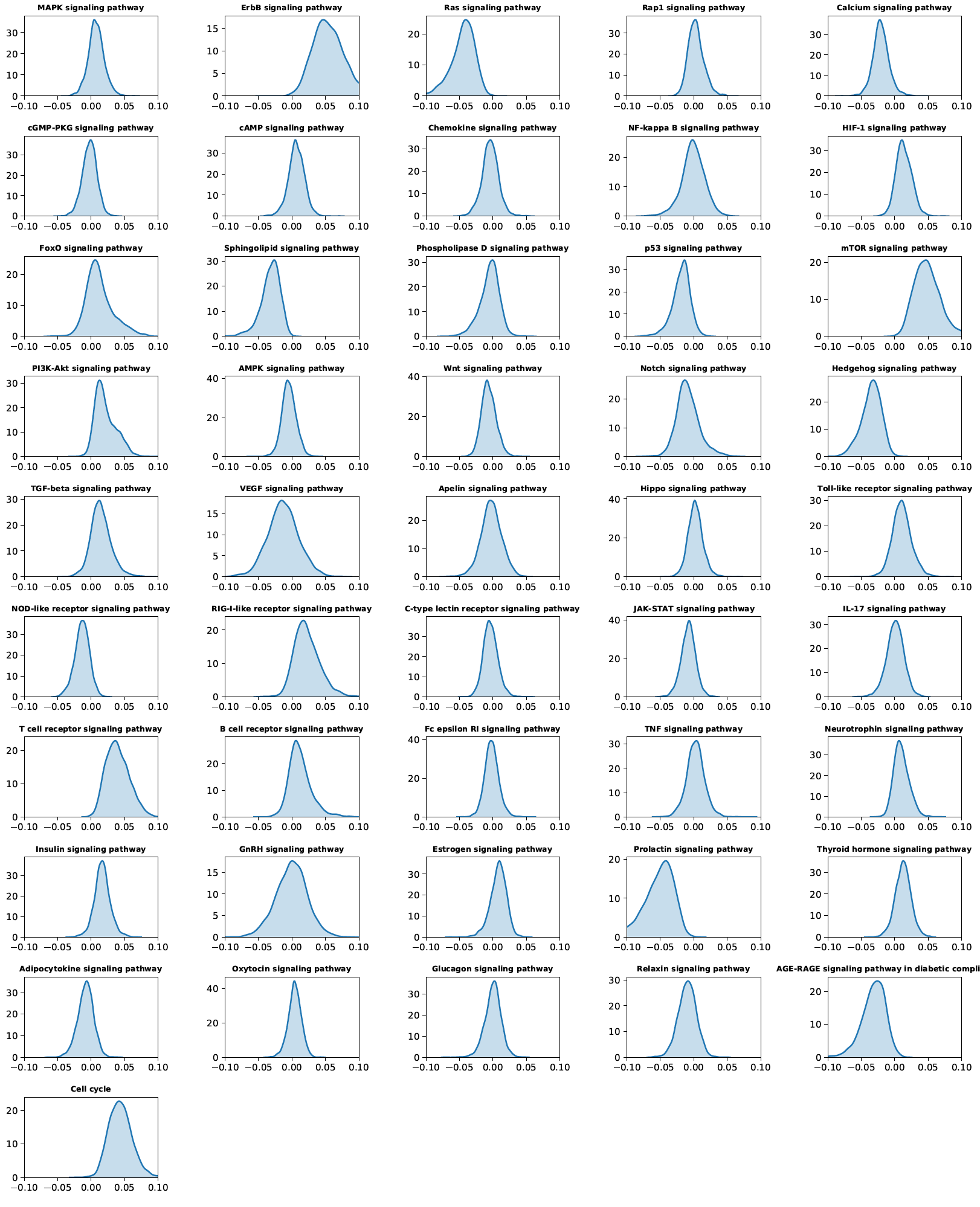


FS.3 Importance scores of the 46 signaling pathways in 3^rd^ split of testing dataset of the 5-fold cross-validation.


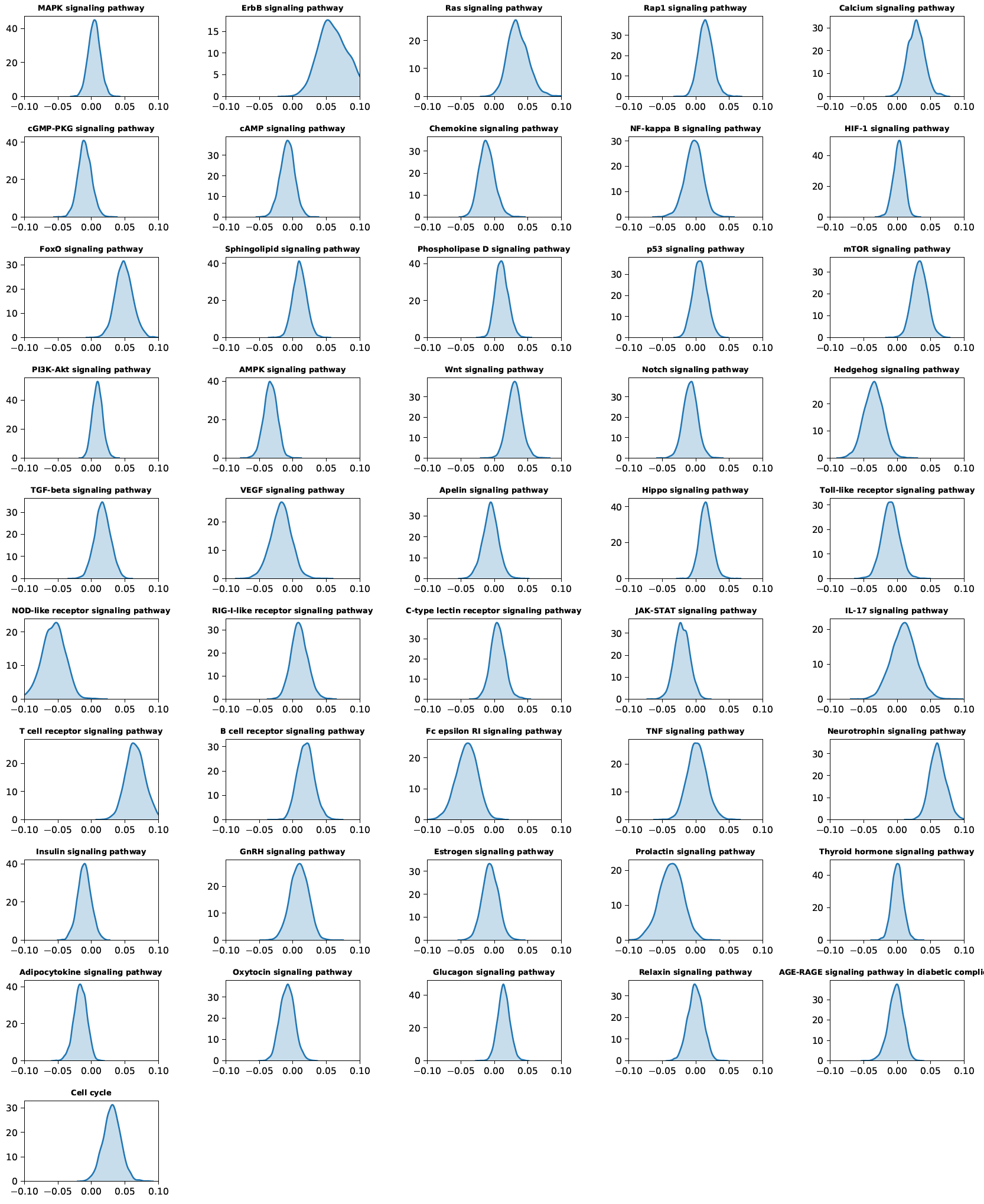


FS.4 Importance scores of the 46 signaling pathways in 4^th^ split of testing dataset of the 5-fold cross-validation.


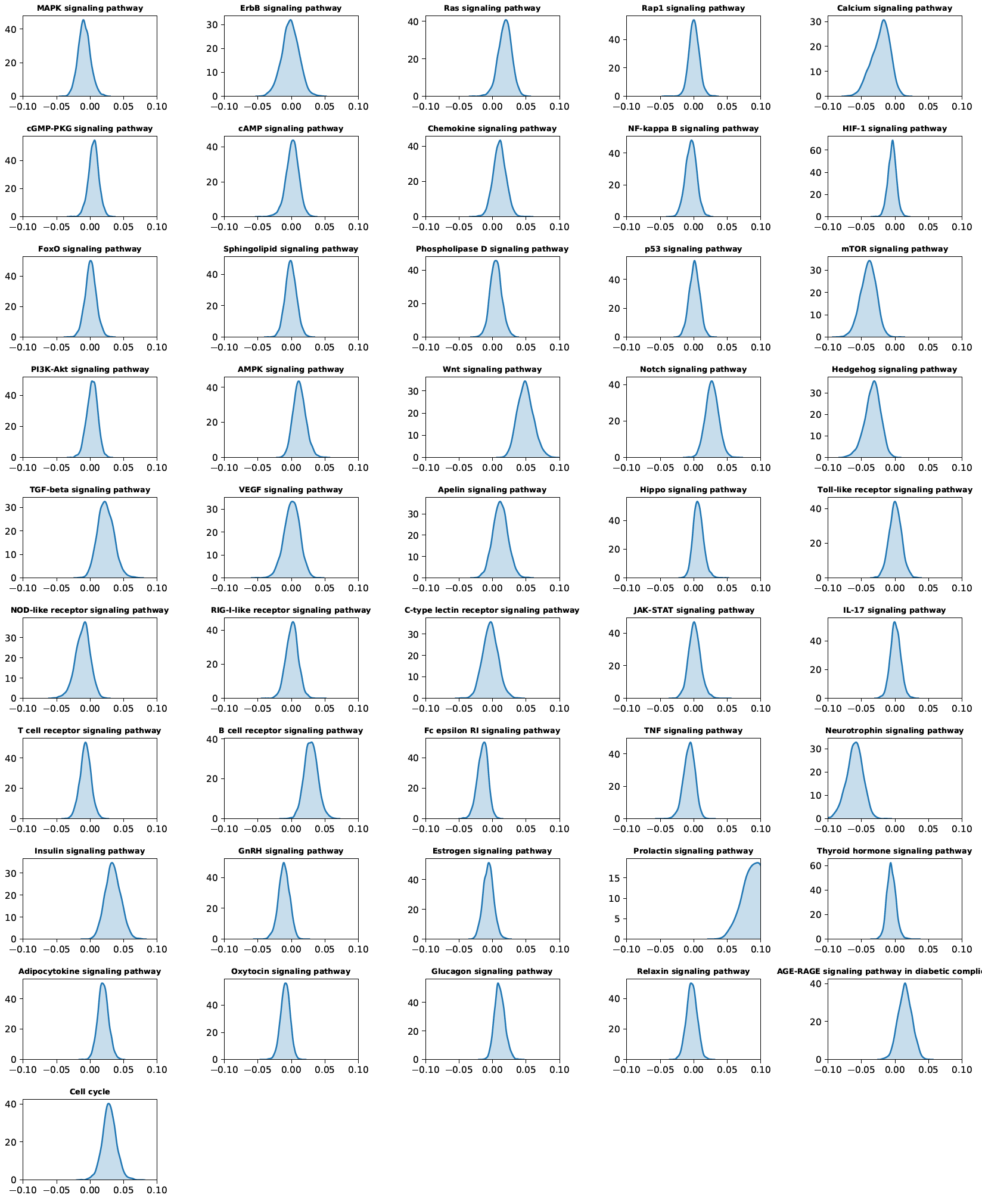


FS.5 Importance scores of the 46 signaling pathways in 5^th^ split of testing dataset of the 5-fold cross-validation.


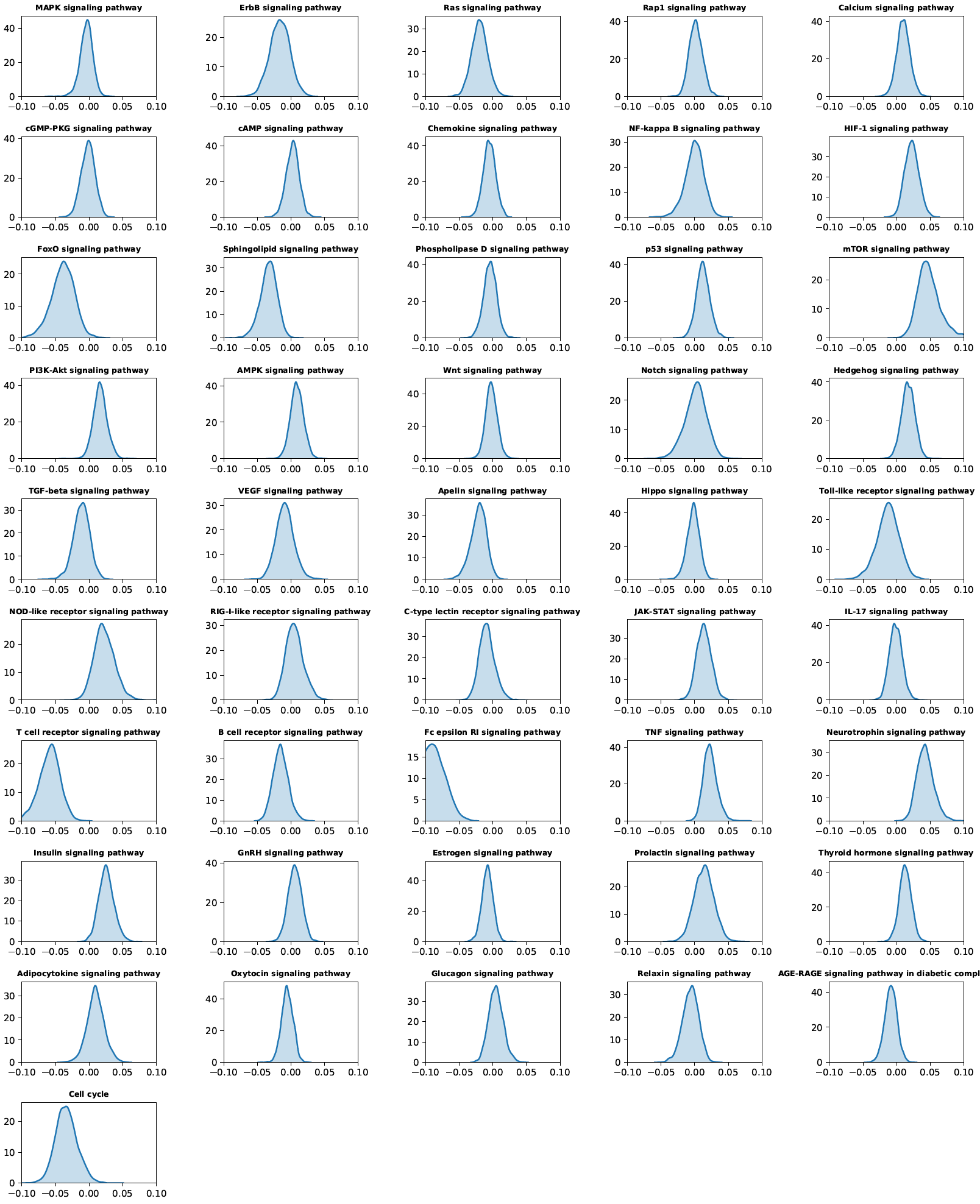

Supplement: Supplementary file 1 [file DataSheet1.zip › Supplementary-Materials_final/SupplementaryFiguresdocx.docx]
